# Rapid and simultaneous detection of *Candida albicans* and *Cryptococcus neoformans* using a duplex real-time RPA assay: a potential point-of-care tool for invasive fungal infections

**DOI:** 10.3389/fcimb.2026.1820215

**Published:** 2026-05-07

**Authors:** Qinglin Ma, Zhihua He, Guikai Duan, Jiahao Wei, Chun Duan, Yan Zhu, Feng Long

**Affiliations:** 1 Department of Clinical Laboratory, Shenzhen Maternity and Child Healthcare Hospital, Southern Medical University, Shenzhen, Guangdong, China; 2 Shenzhen School of Clinical Medicine, Southern Medical University, Shenzhen, Guangdong, China

**Keywords:** *Candida albicans*, *Cryptococcus neoformans*, duplex real-time RPA, invasive fungal infections, molecular diagnostics, point-of-care testing

## Abstract

**Introduction:**

Invasive fungal infections (IFIs) caused by *Candida albicans* and *Cryptococcus neoformans* are associated with high mortality, necessitating rapid and accurate diagnostic tools for timely antifungal therapy.

**Methods:**

We developed and optimized a duplex real-time recombinase polymerase amplification (RPA) assay targeting the internal transcribed spacer (ITS) regions of both fungi. Analytical performance, including specificity, sensitivity, limit of detection (LOD), repeatability and reproducibility, was systematically evaluated. The assay’s diagnostic applicability was assessed using 26 clinical specimens and compared with culture and a duplex qPCR assay.

**Results:**

The assay demonstrated high specificity with no cross-reactivity against non-target fungi or human DNA. The LODs were 559.2 fg/reaction (~34.7 copies) for *C. albicans* and 51.1 fg/reaction (~2.4 copies) for *C. neoformans*, with excellent repeatability and reproducibility (all CV <5%). Performance evaluation on clinical samples showed 100% sensitivity, specificity, positive predictive value (PPV), and negative predictive value (NPV) compared with culture, and exhibited 100% overall agreement with qPCR. The entire process was completed within 20 minutes at 39 °C using a standard real-time PCR instrument.

**Conclusion:**

This rapid, sensitive, and specific duplex real-time RPA assay represents a potential point-of-care testing (POCT) tool for the early and simultaneous diagnosis of two major IFIs-causing pathogens.

## 1 Introduction

Invasive fungal infections (IFIs) are a leading cause of global morbidity and mortality (World Health Organization, 2025), with an estimated 6.55 million annual infections and 3.75 million related deaths worldwide (Wang et al., 2025b). Among global IFIs, invasive candidiasis accounts for nearly 70%, followed by cryptococcosis (20%) and aspergillosis (10%) (Fang et al., 2023). In China, recent epidemiological surveillance data indicate that *Candida albicans* and *Cryptococcus neoformans* remain the predominant pathogens causing invasive candidiasis and cryptococcosis, respectively (Miao et al., 2026). *C. albicans* is often manifesting as bloodstream infections with an in-hospital mortality rate of 30%–40% in critically ill patients (Dangarembizi et al., 2023; Soriano et al., 2023). In contrast, *C. neoformans* is a primary cause of cryptococcal meningitis, which has a mortality rate exceeding 20% despite antifungal therapy (Teekaput et al., 2023) and is highly prevalent in HIV/AIDS patients with low CD4+ T-cell counts (Okafor and Nielsen, 2024; Gifford et al., 2025). The overlapping clinical presentations of these infections, such as fever and neurological symptoms, hinder timely differentiation based on symptoms alone (Maag et al., 2025; Silva et al., 2025). Moreover, co-infections involving both fungi are increasingly reported in severely immunocompromised patients, posing additional diagnostic and therapeutic challenges (Mina et al., 2022; Jafari et al., 2025). Early and accurate pathogen identification is therefore crucial for initiating targeted antifungal therapy, reducing mortality, and preventing unnecessary use of broad-spectrum antifungals (Branda et al., 2025; Wang et al., 2025b).

Conventional diagnostic methods, including fungal culture, microscopy, and serological tests, have inherent limitations (Antinori et al., 2018; Fang et al., 2023). Fungal culture, the gold standard, requires 3–7 days, often delaying targeted therapy (Soriano et al., 2023). Microscopy is rapid but lacks sensitivity, especially for low fungal loads (Maag et al., 2025; Peman and Ruiz-Gaitan, 2025). Serological tests exhibit distinct performance: β-D-glucan (BDG) assay is susceptible to false positivity from multiple sources (e.g., hemodialysis, certain antibiotics) (Im et al., 2024), limiting its role in definitive diagnosis; in contrast, cryptococcal glucuronoxylomannan (GXM) antigen test demonstrates high specificity (Wang et al., 2025a), with only a few cross-reactivity (e.g., *Trichosporon* spp., occasional aspergillosis) that is clinically manageable (Yamashiro-Kanashiro et al., 2025). Molecular techniques like real-time quantitative PCR (qPCR) offer higher sensitivity and specificity but require sophisticated equipment, trained personnel, and relatively long turnaround times (1–2 hours), limiting their use in point-of-care testing (POCT) or resource-limited settings (Fang et al., 2023; Wang et al., 2025b; Sayeli et al., 2026). Nevertheless, neither serological assays nor nucleic acid-based molecular tests can distinguish active infection from colonization or previously treated infection—a limitation that must be considered in clinical interpretation, particularly in patients receiving antifungal therapy (Lass-Florl, 2025).

The clinical need for POCT in IFI diagnosis is particularly pressing in settings where access to sophisticated laboratory infrastructure is limited. As highlighted in a recent comprehensive review on candidemia, delayed diagnosis remains a primary driver of poor outcomes, especially in resource-limited regions and primary care facilities (Dong et al., 2026). POCT-compatible technologies that can be deployed at or near the patient’s bedside have the potential to reduce turnaround times from days to minutes, enabling same-day targeted antifungal therapy and improving antimicrobial stewardship.

Isothermal nucleic acid amplification technologies, particularly recombinase polymerase amplification (RPA), have emerged as promising alternatives for rapid, field-deployable diagnostics (Bi et al., 2025; Wang et al., 2025b). RPA operates at a constant temperature (37–42 °C) and can amplify target DNA within 10–20 minutes using minimal instrumentation (Jin et al., 2025; Sayeli et al., 2026). Real-time RPA combined with fluorescent probes enables real-time monitoring, enhancing detection accuracy (Azizian et al., 2025; Liang et al., 2025). While duplex or multiplex RPA assays have been developed for various pathogens, few studies have focused on simultaneous detection of *C. albicans* and *C. neoformans*, leaving a critical gap in rapid diagnosis of these co-occurring infections (Bi et al., 2025; Zhou et al., 2025).

In this study, we developed and validated a duplex real-time RPA assay for the rapid simultaneous detection of *C. albicans* and *C. neoformans*. By targeting conserved internal transcribed spacer (ITS) regions, we designed species-specific primers and probes, optimized reaction conditions, and systematically evaluated the assay’s analytical performance and clinical applicability. This assay is expected to provide a potential POCT-compatible tool for early diagnosis of IFIs caused by these two fungi, facilitating timely targeted therapy and improving patient outcomes.

## 2 Materials and methods

### 2.1 Ethics statement

This study was approved by the Ethics Committee of the Shenzhen Maternity and Child Healthcare Hospital (approval No. SFYLS[2022]072, approval date 23 December 2022). Written informed consent was waived for residual anonymized specimens used in this study.

### 2.2 Strains, clinical specimens, and DNA extraction

#### 2.2.1 Strain panel for specificity testing

A panel of 20 microbial strains, including reference strains and clinical isolates, was used to assess assay specificity (**Table 1**). Reference strains were obtained from the American Type Culture Collection (ATCC) or the Guangdong Microbial Culture Collection Center (GDMCC). Clinical isolates were collected from Shenzhen Maternity and Child Healthcare Hospital.

**Table 1:** Strains used for specificity evaluation.

Category	Species/strain
*Candida* spp. (n=9)	*C. albicans* ATCC 18804
	*C. tropicalis*
	*C. glabrata* ATCC 2001
	*C. krusei*
	*C. parapsilosis*
	*C. guilliermondii* ATCC 6260
	*C. famata* ATCC 201067
	*C. lusitaniae* ATCC 34449
	*C. dubliniensis*
*Aspergillus* spp. (n=4)	*A. fumigatus* ATCC 96918
	*A. flavus* ATCC 11492
	*A. niger* ATCC 10864
	*A. terreus* ATCC 1012
*Cryptococcus* spp. (n=2)	*C. neoformans* ATCC 208821
	*C. gattii* ATCC MYA-4093
Other Fungi (n=3)	*Trichosporon asahii*
	*Saccharomyces cerevisiae* GDMCC 2.167
	*Talaromyces marneffei*
Bacteria (n=2)	*Staphylococcus aureus*
	*Klebsiella pneumoniae*

Under the “One Fungus, One Name” principle of current taxonomic nomenclature, fungal species with updated accepted names are listed as follows: *Candida glabrata* (accepted name: *Nakaseomyces glabratus*), *Candida krusei* (*Pichia kudriavzevii*), *Candida guilliermondii* (*Meyerozyma guilliermondii*), *Candida famata* (*Debaryomyces hansenii*), *Candida lusitaniae* (*Clavispora lusitaniae*), *Talaromyces marneffei* (formerly *Penicillium marneffei*). Clinical literature retains *Cryptococcus neoformans* and *C. gattii* for familiarity, though their teleomorphs are *Filobasidiella* spp.

#### 2.2.2 Clinical specimens

A total of 26 clinical specimens (one sample from one patient), including 15 blood and 11 cerebrospinal fluid (CSF), were collected from patients with suspected fungal infections who met the 2020 EORTC/MSGERC consensus criteria (Donnelly et al., 2020). Culture was conducted at 25°C and 37°C for up to 4 weeks using the BD BACTEC™ FX Blood Culture System (BD Diagnostics, New Jersey, USA) and standard Sabouraud dextrose agar (Oxoid Limited, Hampshire, UK). Species identification of positive cultures was performed using the MALDI Biotyper System (Bruker Daltonik GmbH, Bremen, Germany) (Ma et al., 2019).

#### 2.2.3 Nucleic acid extraction

Genomic DNA was extracted from strains and clinical specimens using a protocol combining mechanical lysis with glass beads (CapitalBio, Beijing, China) and heat treatment, as described previously (Ma et al., 2019). Crude extracts were further purified using a commercial Fungi Genomic DNA Extraction Kit (Solarbio^®^ Life Sciences, Beijing, China) according to the manufacturer’s instructions. Purified DNA was eluted in 50–200 µL of nuclease-free water, quantified using a Qubit™ 3 Fluorometer (Thermo Fisher Scientific, Wilmington, USA), and stored at −80°C.

### 2.3 Primer and probe design

**Figure 1:**
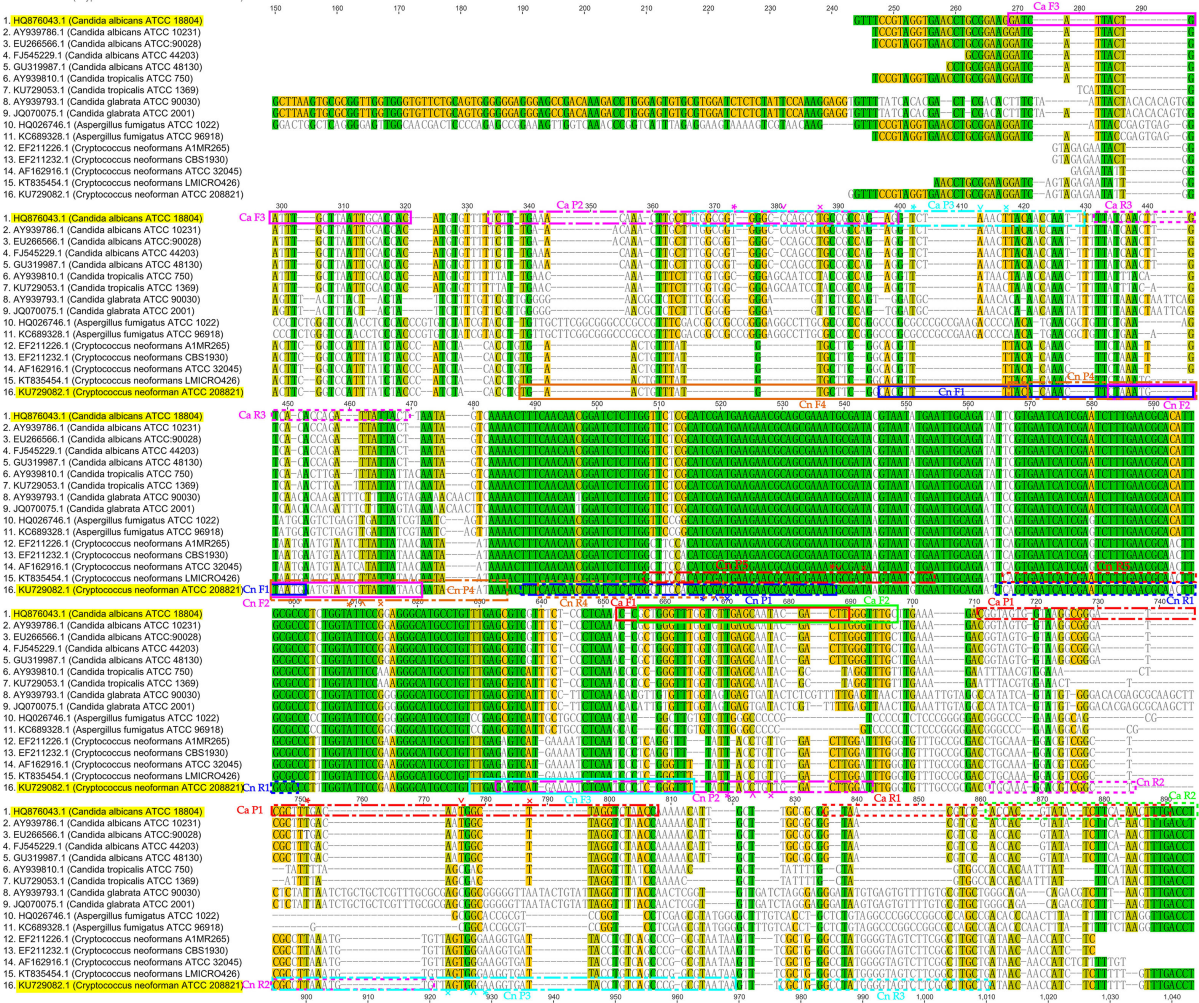
Schematic of the locations of the designed candidate real-time RPA primers and probes within the ITS sequences of various *Candida* species, *Aspergillus fumigatus* and *C. neoformans* strains. Horizontal lines indicate gaps, different colored boxes represent distinct primer-probe sets. Forward primers are marked with solid-line boxes, reverse primers with dashed-line boxes, and Exo probes with dash-dot-line boxes. Within the Exo probes, asterisks (*) indicate fluorophore dye-modified nucleotides, carets (^) represent abasic nucleotide analogs, and crosses (×) denote quencher-modified nucleotides.

The ITS region was chosen as the target. Specifically, the ITS1-5.8S-ITS2 sequences of *C. albicans* (GenBank accession No. HQ876043.1) and *C. neoformans* (GenBank accession No. KU729082.1) served as reference templates. Sequences for 10 C*. albicans* strains, 21 C*. neoformans* strains, and various non-target fungi were obtained from NCBI GenBank and aligned using Geneious^®^ 6.1.3 and DNAMAN V6.0.3.99 to identify conserved regions. Candidate primers and probes were designed following the TwistAmp^®^ design manual (TwistDx, Cambridge, UK) and are illustrated in **Figure 1**. All oligonucleotides were synthesized by Sangon Biotech (Shanghai, China).

### 2.4 Duplex real-time RPA assay and optimization

The RPA assay was performed in a final volume of 50 µL using the TwistAmp^®^ Liquid Exo kit (TwistDx™ Limited, UK). The reaction mixture comprised 20x Core Reaction mix, 50x Exo, dNTPs, 10x Probe E-mix, 2x Reaction Buffer, primers and probes, template DNA, nuclease-free water, and MgOAc solution. Amplification and real-time fluorescence detection were conducted on a LightCycler^®^ 96 System (Roche Diagnostics, Branchburg, NJ, USA) under the following conditions: 39°C for 20 minutes (60 cycles), with fluorescence acquisition every 20 seconds (Ma et al., 2017, 2019). A non-template control (NTC, nuclease-free water instead of template DNA) was set in each run to monitor reagent contamination and non-specific amplification.

Key parameters were systematically optimized, including primer-probe combinations, primer and probe concentrations/ratios, and Mg^2+^ concentration. A sample was scored as positive if it produced a characteristic exponential amplification curve with fluorescence signal clearly exceeding the baseline level of negative controls.

### 2.5 Analytical specificity, sensitivity, repeatability, and reproducibility

Specificity was evaluated using excessive amounts of genomic DNA from *Homo sapiens* and non-target pathogens listed in **Table 1**. Sensitivity was determined using serial tenfold dilutions of target genomic DNA. Each dilution was tested in triplicate for preliminary assessment and in ten independent runs for LOD determination. The limit of detection (LOD) at 95% detection probability was calculated using probit regression analysis with IBM SPSS Statistics (version 21.0). As a common design for the validation of isothermal amplification assays (Sun et al., 2025; Zhou et al., 2025), intra-assay precision (repeatability) was assessed by analyzing three different DNA concentrations in triplicate within a single run; inter-assay precision (reproducibility) was evaluated by testing the same concentrations in triplicate across three independent runs on separate days; the coefficients of variation (CV) for cycle threshold (Ct) values were calculated for both precision measures.

### 2.6 Performance evaluation on clinical samples and statistical analysis

To evaluate the diagnostic performance, 26 clinical specimens were tested in parallel using a previously reported duplex qPCR (Wen et al., 2020) and the newly developed duplex real-time RPA assay. The results were interpreted blindly and then compared with those of fungal culture. Diagnostic parameters such as sensitivity and specificity were calculated using the online diagnostic test evaluation calculator (https://www.medcalc.org/en/calc/diagnostic_test.php). A method comparison study was conducted, and the concordance between qPCR and RPA was quantified using Cohen’s *kappa* (*κ*) statistic. According to the Landis and Koch interpretation scale (Landis and Koch, 1977), *kappa* values of 0.81–1.00 represent almost perfect agreement (Vanbelle et al., 2024). The *P*-value (<0.05) indicates that the observed agreement is significantly greater than what would be expected by chance alone, rejecting the null hypothesis of no agreement beyond chance. All other statistical analyses were performed using GraphPad Prism (version 8.4) and SPSS (version 21.0).

## 3 Results

### 3.1 Screening of optimal primer-probe combinations

**Figure 2:**
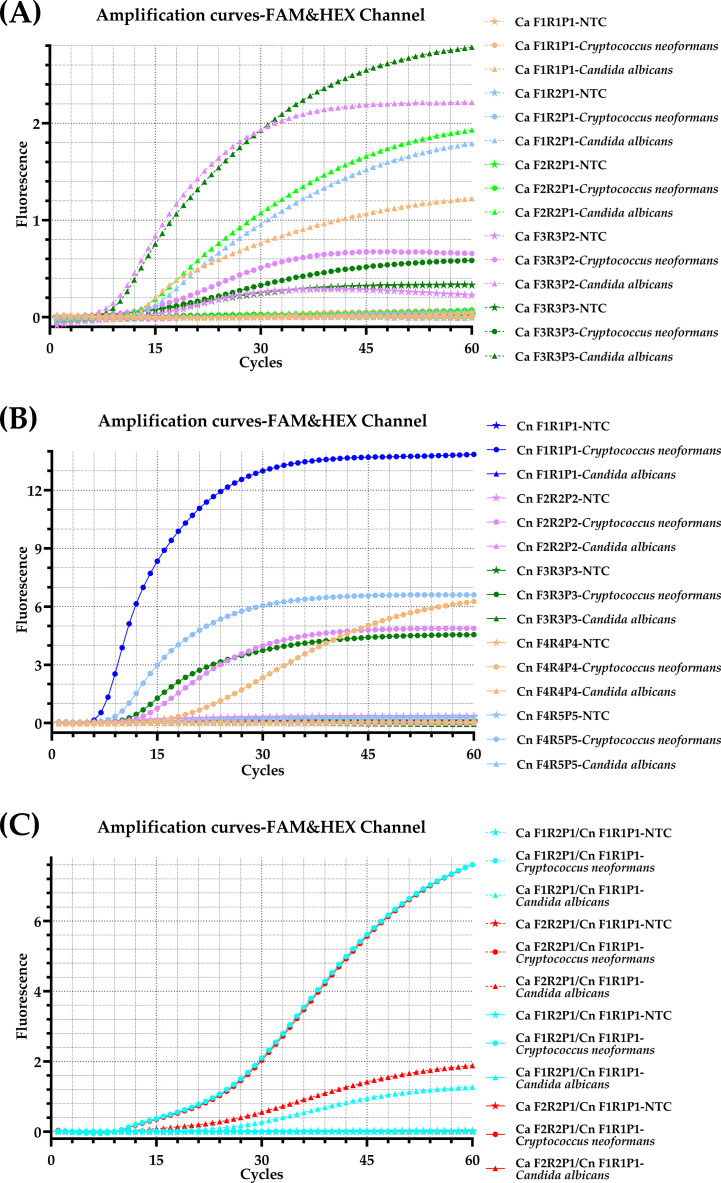
Screening of optimal primer-probe combination. Representative amplification curves of real-time RPA assays using primer-probe sets shown in **Figure 1**. Different colors correspond to distinct primer-probe sets; dashed lines indicate FAM channel; solid lines represent HEX channel; star symbols (☆) denote non-template control (NTC); filled circles (●) represent *C. neoformans* genomic DNA; triangles (△) indicate *C. albicans* genomic. **(A)** Screening of specific primer-probe sets for *C. albicans*; **(B)** Screening for *C. neoformans*; **(C)** Screening of duplex combinations. FAM, 6-carboxyfluorescein; HEX, 5-hexachlorofluorescein.

Following alignment of ITS sequences, several candidate primer pairs and probes were designed (**Figure 1**). In initial singleplex screening, primer-probe sets Ca F2R2P1 and Ca F1R2P1 (FAM-labeled) for *C. albicans* exhibited favorable amplification efficiency and specificity (**Figure 2A**). For *C. neoformans*, Cn F1R1P1 (HEX-labeled) showed the highest amplification efficiency with no cross-reactivity (**Figure 2B**). Subsequent duplex screening confirmed that the combination Ca F2R2P1 and Cn F1R1P1 demonstrated superior amplification efficiency for both targets while maintaining excellent specificity and low background (**Figure 2C**). This optimal combination (**Table 2**) was used in all further assays.

**Table 2:** Primers and probes used in duplex real-time RPA assay.

Target	Name	Sequence (5’→3’)	Product
*C. albicans*	Ca F2	GCTGGGTTTGGTGTTGAGCAATACGACTTGGGTTTGC	147 bp
	Ca R2	AGGTCAAAGTTTGAAGATATACGTGGT	
	Ca P1	CGGTAGTGGTAAGGCGGGATCGCTT[FAM-dT]GACAA[dSpacer]GGC[BHQ1-dT]TAGGTCTAACC[C3 Spacer]	
*C. neoformans*	Cn F1	CACGTTTTACACAAACTTCTAAATGTAATG	173 bp
	Cn R1	GCGCAAGTTGCGTTCAAAGATTCGATGATTCACTGA	
	Cn P1	TTTCAACAACGGATCTCTTGGCTTCCACA[HEX-dT]C[dSpacer]A[BHQ1-dT]GAAGAACGCAGCGAAAT[C3 Spacer]	

FAM-dT, thymidine conjugated with 6-carboxyfluorescein; HEX-dT, thymidine conjugated with 5-hexachlorofluorescein; BHQ1-dT, thymidine conjugated with black hole quencher 1; dSpacer, abasic nucleotide analog; C3 Spacer, 3’-modification phosphate group to inhibit elongation.

### 3.2 Optimization of duplex real-time RPA conditions

**Figure 3:**
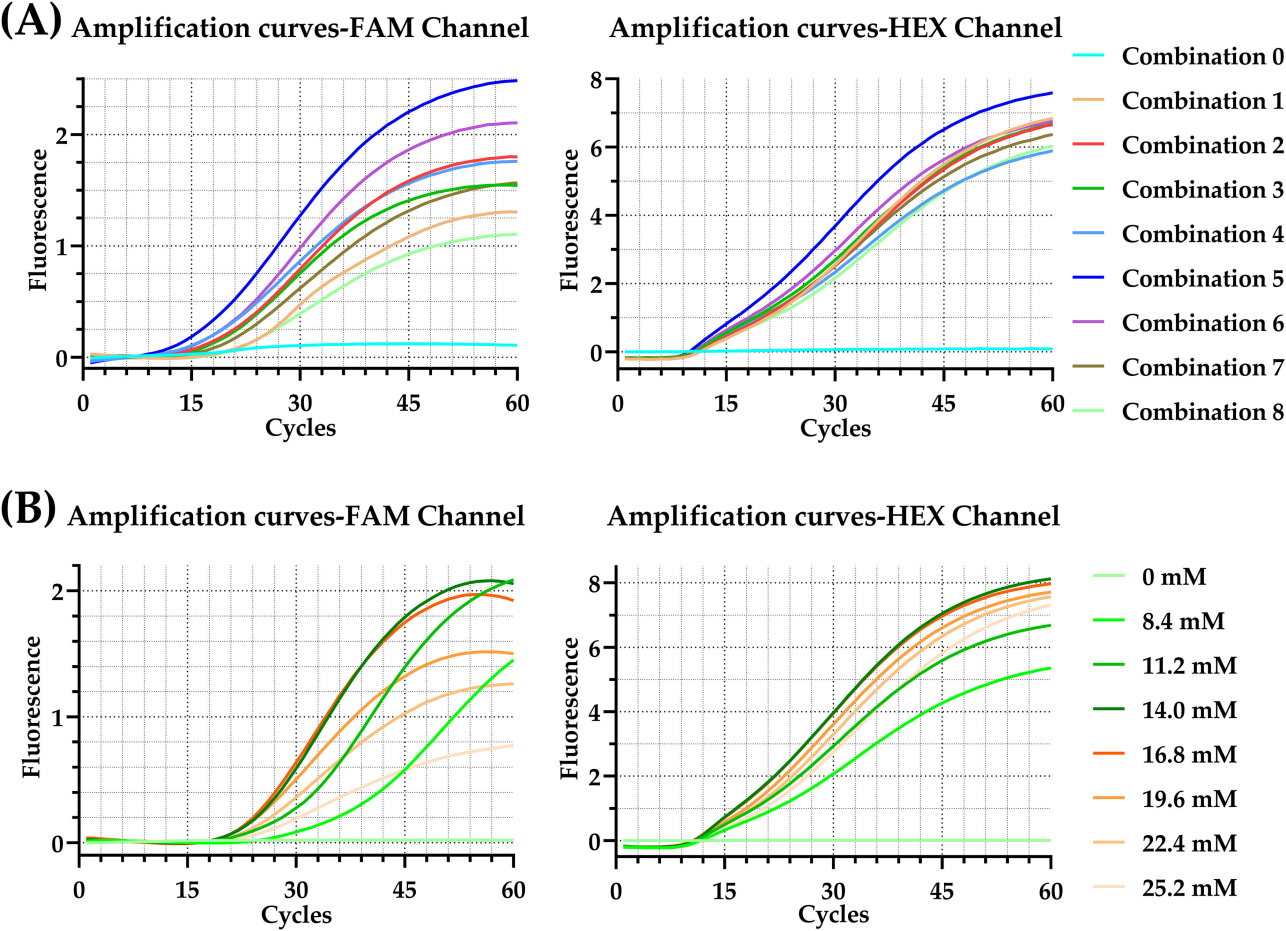
Optimization of reaction conditions for the duplex real-time RPA assay. Representative amplification curves in different colors represent different primer-probe concentration ratios **(A)** or different Mg^2+^ concentrations **(B)**. FAM and HEX channels correspond to *C. albicans* and *C. neoformans*, respectively. FAM, 6-carboxyfluorescein; HEX, 5-hexachlorofluorescein.

Optimization of primer-probe concentration ratios (**Table 3**) revealed that the experimental combination featuring 760 nM primers (F:R = 1:1) and 100 nM probe for *C. albicans*, and 560 nM primers (F:R = 1:1) and 80 nM probe for *C. neoformans*, yielded the highest amplification efficiency (**Figure 3A**). Mg^2+^ concentration optimization demonstrated that fluorescence intensity peaked at 14.0 mM and 16.8 mM; 14.0 mM was selected to minimize potential nonspecific amplification (**Figure 3B**). Final optimal conditions were established as 39°C for 20 minutes with the aforementioned primer/probe concentrations and 14.0 mM Mg^2+^.

**Table 3:** Primers and probes concentration ratios tested in **Figure 3A**.

Target	Component	The experimental combinations (nM)								
		0	1	2	3	4	5	6	7	8
*C. albicans*	Ca F2	0	400	390	380	370	380	370	360	350
	Ca R2	0	400	390	380	370	380	370	360	350
	Ca P1	0	100	120	140	160	100	120	140	160
*C. neoformans*	Cn F1	0	260	260	260	260	280	280	280	280
	Cn R1	0	260	260	260	260	280	280	280	280
	Cn P1	0	80	80	80	80	80	80	80	80

### 3.3 Analytical specificity

**Figure 4:**
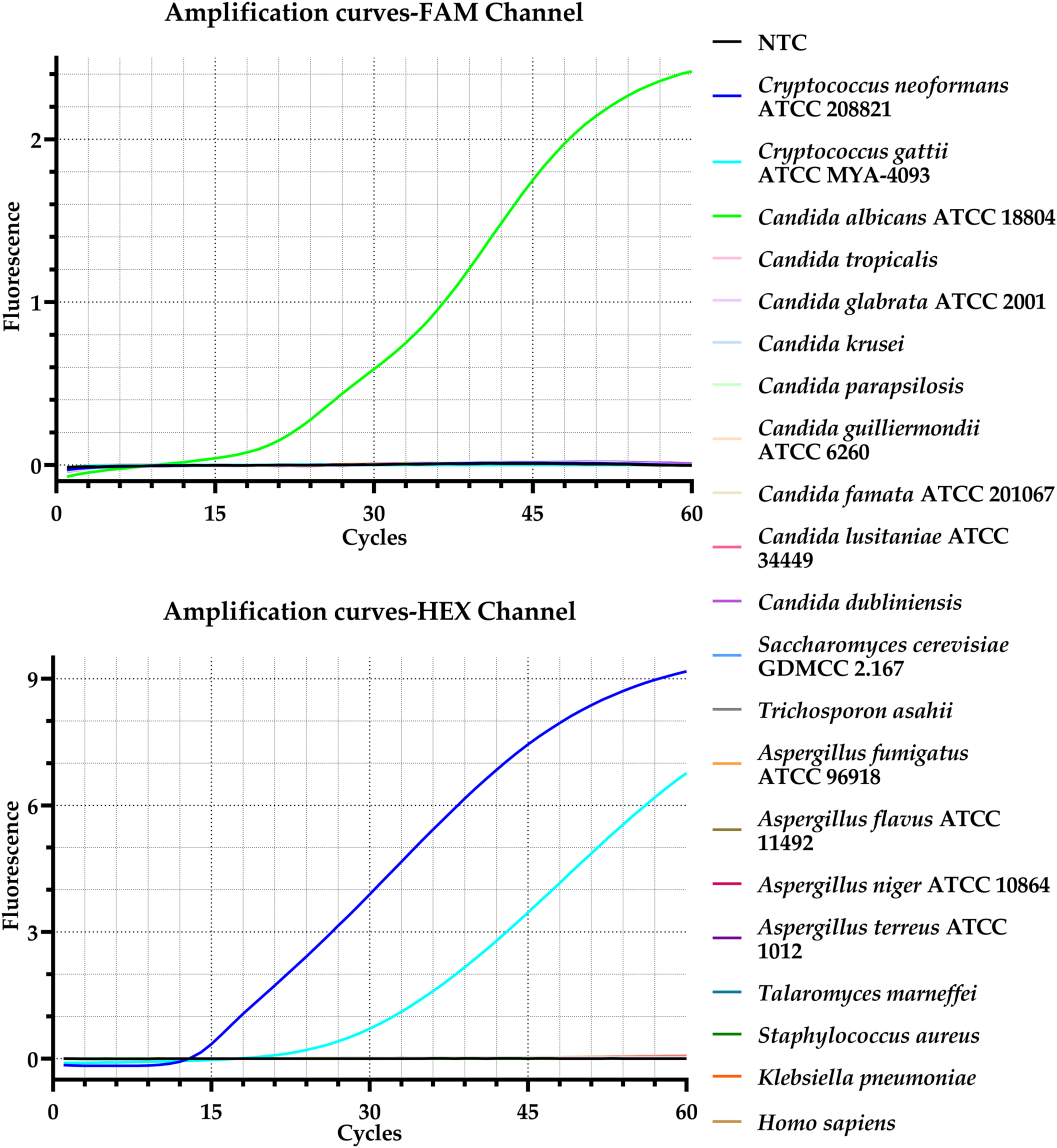
Specificity analysis of the duplex real-time RPA assay. Probes for *C. albicans* and *C. neoformans* were labeled with FAM and HEX, respectively. Different colors correspond to distinct species. All strains were clinical isolates unless otherwise specified. FAM, 6-carboxyfluorescein; HEX, 5-hexachlorofluorescein; NTC, non-template control; ATCC, American Type Culture Collection; GDMCC, Guangdong Microbial Culture Collection Center.

Positive amplification signals were observed for *C. albicans* in the FAM channel, and for *C. neoformans* and notably *Cryptococcus gattii* (ATCC MYA-4093) in the HEX channel (**Figure 4**). This cross-reactivity is attributed to the high sequence homology (>95%) in the ITS regions of *C. neoformans* and *C. gattii*, which are taxonomically closely related and are even considered a species complex. No cross-reactivity was detected with *Homo sapiens* genomic DNA (tested at concentrations up to 100 ng/reaction) or any non-target pathogens, confirming that the assay is highly specific and suitable for direct testing of clinical specimens with human DNA background.

### 3.4 Analytical sensitivity

**Figure 5:**
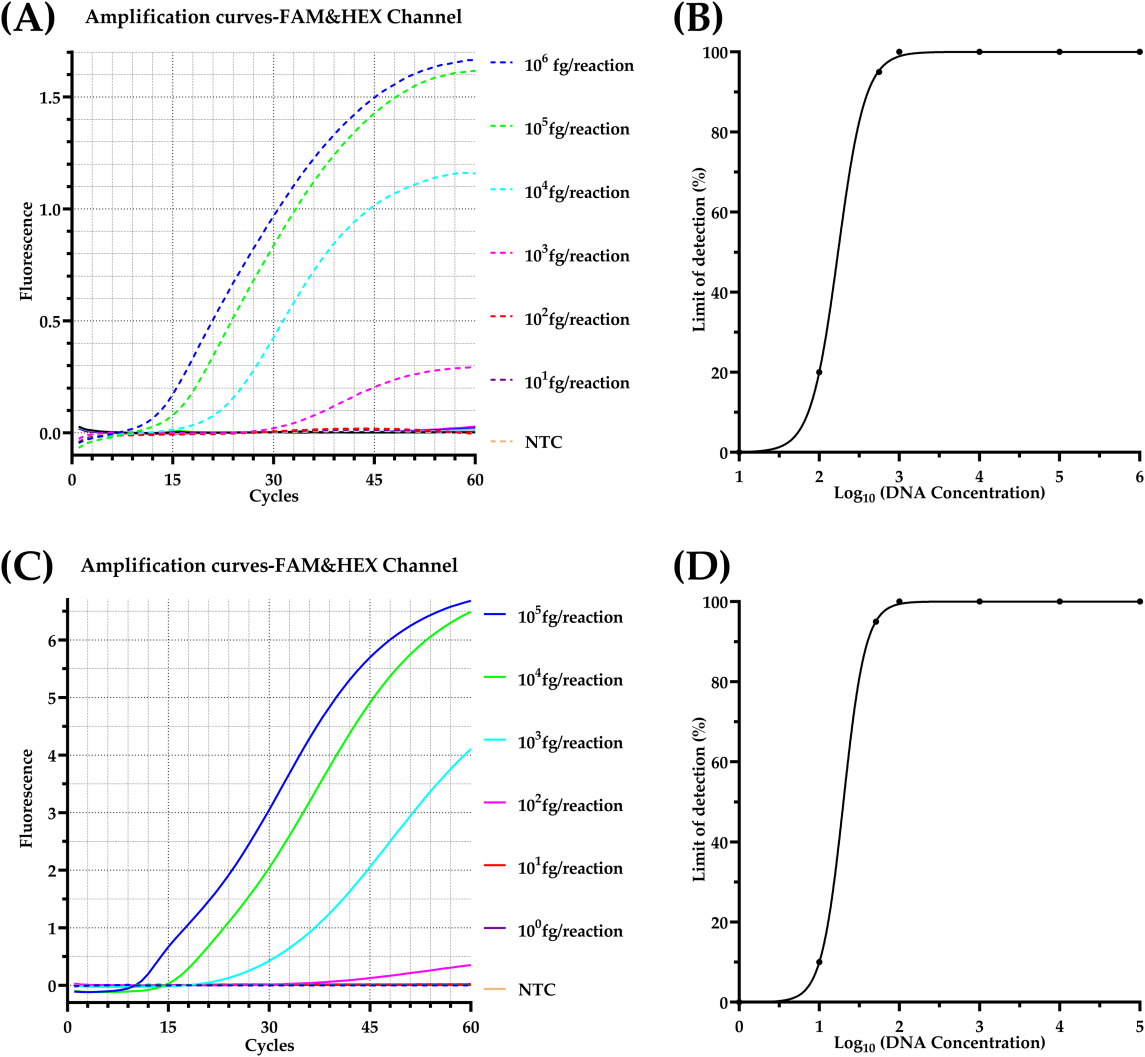
Sensitivity analysis of the duplex real-time RPA assay. Representative amplification curves in different colors correspond to distinct template concentration and non-template control (NTC); dashed lines indicate FAM channel, while solid lines represent HEX channel. **(A)** Analytical sensitivity for *C. albicans*; **(B)** Probit regression analysis for *C. albicans*; **(C)** Analytical sensitivity for *C. neoformans*; **(D)** Probit regression analysis for *C. neoformans*. FAM, 6-carboxyfluorescein; HEX, 5-hexachlorofluorescein.

The visual limit of detection was 10^3^ fg/reaction for *C. albicans* (**Figure 5A**) and 10^2^ fg/reaction for *C. neoformans* (**Figure 5C**). Probit regression analysis determined the LOD at 95% probability to be 559.2 fg/reaction (~34.7 copies/reaction) for *C. albicans* (**Figure 5B**) and 51.1 fg/reaction (~2.4 copies/reaction) for *C. neoformans* (**Figure 5D**). DNA copy number was calculated using the formula: copies/reaction = [genomic DNA concentration (fg/μL)×10^-15^×6.02×10^23^]/[DNA length (bp)×660].

### 3.5 Repeatability and reproducibility

All intra- and inter-assay CVs for Ct values were below 5% for both targets across all tested concentrations (**Table 4**), demonstrating excellent repeatability and reproducibility.

**Table 4:** Repeatability and reproducibility of the duplex real-time RPA assay.

Templates (fg/reaction)		Repeatability		Reproducibility	
		Ct (mean ± SD)	CV (%)	Ct (mean ± SD)	CV (%)
*C. albicans*	10^5^	11.99 ± 0.15	1.25	12.54 ± 0.51	4.10
	10^4^	20.16 ± 0.11	0.56	21.11 ± 1.02	4.83
	10^3^	31.36 ± 0.33	1.04	31.12 ± 0.21	0.69
*C. neoformans*	10^4^	12.68 ± 0.50	3.94	12.83 ± 0.25	1.98
	10^3^	18.77 ± 0.56	2.96	18.58 ± 0.38	2.07
	10^2^	34.42 ± 1.47	4.28	34.84 ± 0.94	2.69

### 3.6 Performance evaluation on clinical samples

Compared with fungal culture, the duplex RPA assay demonstrated 100% sensitivity, specificity, positive predictive value (PPV), and negative predictive value (NPV) in clinical specimens, with diagnostic performance completely comparable to that of qPCR (**Table 5**). Meanwhile, RPA exhibited 100% overall agreement with qPCR for the detection of *C. albicans* and *C. neoformans*, with a *Kappa* value of 1.000 and a *P*-value <0.001 for each of the two pathogens (**Table 6**).

**Table 5:** Estimation of diagnostic validity of duplex RPA with clinical specimens.

Assay		Culture		Performance characteristics (%)			
		Positive	Negative	Sensitivity	Specificity	Positive predictive value^*^	Negative predictive value^*^
RPA	Positive	14	0	100% ^a^	100% ^b^	100% ^c^	100% ^d^
	Negative	0	12				
qPCR	Positive	14	0	100% ^a^	100% ^b^	100% ^c^	100% ^d^
	Negative	0	12				

*These values are dependent on disease prevalence. CI, confidence interval. ^a^95% CI: 76.84%–100.00%; ^b^95% CI: 73.54%–100.00%; ^c^95% CI: 76.84%–100.00%; ^d^95% CI: 73.54%–98.19%.

**Table 6:** Comparative analysis of duplex RPA versus duplex qPCR using clinical samples.

Target	qPCR	RPA			Overall agreement	*Kappa*	*P*-value
		Positive	Negative	Total			
*C. albicans*	Positive	8	0	8	100.00%	1.000	<0.001
	Negative	0	18	18			
	Total	8	18	26			
*C. neoformans*	Positive	6	0	6	100.00%	1.000	<0.001
	Negative	0	20	20			
	Total	6	20	26			

## 4 Discussion

IFIs caused by *C. albicans* and *C. neoformans* pose significant threats to immunocompromised populations, with high mortality often attributed to delayed or inaccurate diagnosis (Hoenigl et al., 2021; Sedik et al., 2024; Pechacek and Lionakis, 2026). Conventional diagnostics, such as culture and microscopy, are time-consuming or lack sensitivity. Although qPCR offers accuracy, its reliance on sophisticated instrumentation and longer turnaround times limits its application in POCT or resource-limited settings (Arastehfar et al., 2021; Fang et al., 2023). Therefore, rapid, simple, and accurate detection platforms are urgently needed to improve clinical management of IFIs.

In this study, we developed and validated a duplex real-time RPA assay for simultaneous detection of *C. albicans* and *C. neoformans*. By targeting the conserved ITS region, we identified an optimal primer-probe combination that demonstrated excellent specificity, with no detectable cross-reactivity against a broad panel of non-target fungi and bacteria (**Figure 4**). This high specificity aligns with other RPA-based assays (Zhou et al., 2025) and is crucial for preventing false-positive results in complex clinical specimens (Cruciani et al., 2019).

The assay exhibited cross-reactivity with *C. gattii*, owing to the high sequence identity (>95% homology) in the ITS regions between these two closely related species, which are even regarded as a species complex (Passer et al., 2019; Oliveira et al., 2021; Beardsley et al., 2022). While this limits species-level discrimination within the *C. gattii*/*neoformans* complex, it effectively broadens the assay’s capability to detect pathogenic *Cryptococcus* species. This may be an acceptable or even advantageous feature for initial screening, where the primary goal is to rule out cryptococcosis. For definitive speciation, subsequent analysis with a species-specific assay would be recommended. Future primer/probe designs could explore single-nucleotide polymorphisms to potentially resolve this cross-reactivity while maintaining high sensitivity.

The assay’s analytical sensitivity (LOD of ~34.7 copies for *C. albicans* and ~2.4 copies for *C. neoformans*) is comparable to or surpasses that of duplex qPCR (Wen et al., 2020) and singleplex RPA assays (Azizian et al., 2025). This high sensitivity, coupled with excellent precision (CV<5%, **Table 4**), underscores the efficiency and reliability of our optimized system (Jenks et al., 2023). Following CLSI EP05 guidelines, future studies with larger sample sizes (e.g., n=10 per concentration for intra-assay precision and 5–10 independent runs on separate days for inter-assay precision) would provide more definitive precision data.

Performance evaluation across 26 specimens demonstrated excellent diagnostic parameters compared with gold standard (fungal culture, **Table 5**) and a statistically highly significant level of concordance with duplex qPCR (**Table 6**). No *C. gattii* infections were encountered in this clinical cohort (**Supplementary Table 1**), which is consistent with the epidemiological distribution of *Cryptococcus* species in our region. Therefore, the cross-reactivity with *C. gattii* observed in analytical testing did not affect clinical specificity in this cohort, although it would be expected to yield positive results for *C. gattii* if such specimens were encountered.

The assay offers rapid turnaround, with amplification completed in just 20 minutes at 39 °C. However, the total time-to-result, including DNA extraction (~45 minutes), is approximately 65–70 minutes. While this is still faster than conventional culture (3–7 days) and comparable to expedited qPCR protocols (Sayeli et al., 2026), it falls short of true POCT standards. It should also be noted that in routine laboratory practice, batching of specimens may introduce additional delays; the 20-minute amplification time represents instrument run time only, not the total laboratory turnaround time. For cryptococcosis diagnosis, the cryptococcal GXM lateral flow assay (CrAg LFA) represents an established POCT benchmark, with a total turnaround time of <15 minutes, minimal equipment requirements, and excellent diagnostic performance (sensitivity >99% in CSF) (Chang et al., 2024). In comparison, our molecular assay, while offering the advantage of simultaneous detection of both *C. albicans* and *C. neoformans*, requires a longer total time and more complex sample processing. Therefore, in settings where cryptococcosis is the primary clinical concern and rapid diagnosis is critical (e.g., HIV-associated meningitis), the CrAg LFA remains the test of choice. Our duplex RPA assay may be more suitable for settings where both candidiasis and cryptococcosis are included in the differential diagnosis, or as a confirmatory or adjunctive test.

Its compatibility with standard real-time PCR instruments provides an immediate pathway for adoption in centralized labs. More importantly, the isothermal nature of RPA obviates the need for complex thermal cyclers (Jin et al., 2025), rendering it compatible with compact, portable fluorometers (Sun et al., 2025). Furthermore, compared to other isothermal techniques such as loop-mediated isothermal amplification, RPA functions at a lower and more easily sustained temperature range (37–42°C), further reducing instrumental complexity (Tan et al., 2022). This compatibility underscores a clear pathway toward deploying a truly decentralized point-of-care diagnostic test in resource-limited settings, directly addressing a critical diagnostic gap for IFIs where treatment delays contribute to poor outcomes (Wang et al., 2025b; Zhou et al., 2025).

Despite the promising performance demonstrated in this study, several limitations must be acknowledged, particularly regarding the assay’s applicability as a true POCT tool. First, all validation experiments were performed on a standard laboratory real-time PCR instrument (LightCycler^®^ 96 System) using a 50 µL reaction volume. While compatible with the assay, this setup does not represent a true POCT platform, and neither the reaction system nor the reagent formulations have been optimized for miniaturization or integration into microfluidic cartridges. Additionally, the assay has not yet been validated on portable, battery-operated fluorometers suitable for decentralized testing. Second, despite good analytical performance, the clinical validation cohort was of small size (n=26). Future larger-scale multi-center studies with more diverse specimen types (e.g., tissue biopsies) and patient populations pediatricare needed to further substantiate clinical utility. Third, like most molecular assays, the current method requires an upfront DNA extraction step, which adds to the total turnaround time and limits its deployment in truly resource-limited settings. Finally, the assay currently targets only two major fungal pathogens. Given the diverse etiology of IFIs, expanding the multiplexing capacity to include other clinically relevant fungi (e.g., *Aspergillus* species, *Candida glabrata*) would enhance its diagnostic comprehensiveness (Slepcanova et al., 2025; Tsuchido, 2025). To address these limitations and advance toward a field-deployable POCT tool, future work will focus on: (1) optimizing a reduced-volume (e.g., 10–20 µL) reaction system compatible with microfluidic platforms; (2) validating the assay on portable real-time fluorometers in resource-limited settings; and (3) integrating simplified nucleic acid extraction protocols and lyophilized reagent storage to enable true sample-to-answer detection; and (4) expanding the multiplex detection range and conducting large-scale multi-center clinical verification to fully translate this laboratory-based assay into a field-deployable POCT tool for invasive fungal infections (Ma et al., 2019; Liang et al., 2025).

In conclusion, we have developed a novel duplex real-time RPA assay enabling the rapid, specific, sensitive, and simultaneous detection of *C. albicans* and *C. neoformans*. By integrating the speed and instrumental simplicity of isothermal amplification with the specificity and multiplexing capability of real-time fluorescence detection, this assay achieves excellent analytical and clinical performance. These results position it as a potential point-of-care diagnostic tool for early accurate diagnosis of invasive fungal infections, particularly in settings requiring rapid turnaround to guide timely antifungal therapy.

## Data Availability

The original contributions presented in the study are included in the article/**Supplementary Material**. Further inquiries can be directed to the corresponding authors.
