# Supplementary material for: Rapid and simultaneous detection of Candida albicans and Cryptococcus neoformans using a duplex real-time RPA assay: a potential point-of-care tool for invasive fungal infections
Source: Front Cell Infect Microbiol. 2026 May 7;16:1820215. doi: 10.3389/fcimb.2026.1820215 (PMC13189755; doi:10.3389/fcimb.2026.1820215)
Supplement: Supplementary file 1 [file Table1.docx]

# Supplementary Tables

**TABLE S1 Raw results of 26 clinical specimens.**

| **No.** | **Specimens** | **Source** | **Culture** | **qPCR*** | **RPA** |
| --- | --- | --- | --- | --- | --- |
| 1 | Peripheral Venous Blood | NICU | *C. albicans* | *C. albicans* | *C. albicans* |
| 2 | Peripheral Venous Blood | NICU | *C. albicans* | *C. albicans* | *C. albicans* |
| 3 | Peripheral Venous Blood | NICU | *C. albicans* | *C. albicans* | *C. albicans* |
| 4 | Peripheral Venous Blood | NICU | *C. albicans* | *C. albicans* | *C. albicans* |
| 5 | Umbilical Cord Blood | NICU | *C. albicans* | *C. albicans* | *C. albicans* |
| 6 | Umbilical Cord Blood | NICU | *C. albicans* | *C. albicans* | *C. albicans* |
| 7 | Umbilical Cord Blood | NICU | *C. albicans* | *C. albicans* | *C. albicans* |
| 8 | Umbilical Cord Blood | NICU | *C. albicans* | *C. albicans* | *C. albicans* |
| 9 | Cerebrospinal Fluid | PICU | *C. neoformans* | *C. neoformans* | *C. neoformans* |
| 10 | Cerebrospinal Fluid | PICU | *C. neoformans* | *C. neoformans* | *C. neoformans* |
| 11 | Cerebrospinal Fluid | PICU | *C. neoformans* | *C. neoformans* | *C. neoformans* |
| 12 | Cerebrospinal Fluid | PICU | *C. neoformans* | *C. neoformans* | *C. neoformans* |
| 13 | Cerebrospinal Fluid | PICU | *C. neoformans* | *C. neoformans* | *C. neoformans* |
| 14 | Cerebrospinal Fluid | NICU | *C. neoformans* | *C. neoformans* | *C. neoformans* |
| 15 | Peripheral Venous Blood | Gynecology | *C. glabrata* | No specific melting peaks | No amplification signals |
| 16 | Peripheral Venous Blood | NICU | *C. glabrata* | No specific melting peaks | No amplification signals |
| 17 | Peripheral Venous Blood | NICU | *C. parapsilosis* | No specific melting peaks | No amplification signals |
| 18 | Umbilical Cord Blood | NICU | *C. glabrata* | No specific melting peaks | No amplification signals |
| 19 | Umbilical Cord Blood | NICU | *C. glabrata* | No specific melting peaks | No amplification signals |
| 20 | Umbilical Cord Blood | NICU | *C. glabrata* | No specific melting peaks | No amplification signals |
| 21 | Umbilical Cord Blood | NICU | *C. glabrata* | No specific melting peaks | No amplification signals |
| 22 | Cerebrospinal Fluid | PICU | No fungal growth | No specific melting peaks | No amplification signals |
| 23 | Cerebrospinal Fluid | PICU | No fungal growth | No specific melting peaks | No amplification signals |
| 24 | Cerebrospinal Fluid | PICU | No fungal growth | No specific melting peaks | No amplification signals |
| 25 | Cerebrospinal Fluid | NICU | No fungal growth | No specific melting peaks | No amplification signals |
| 26 | Cerebrospinal Fluid | Neonatology | No fungal growth | No specific melting peaks | No amplification signals |

NICU: neonatal intensive care unit; PICU: pediatric intensive care unit. * Only specific probes targeting *C. albicans* and *C. neoformans* were employed; hence, *C. glabrata* and *C. parapsilosis* could not be detected.
